# Lipidomic data uncover extensive heterogeneity in phosphatidylcholine structural variants in HepG2 cells

**DOI:** 10.1016/j.dib.2019.104608

**Published:** 2019-10-04

**Authors:** Yolanda Chico, Beatriz Abad-García, Begoña Ochoa, María José Martínez

**Affiliations:** a Lipids & Liver Research Group, Department of Physiology, Faculty of Medicine and Nursing, University of the Basque Country UPV/EHU, Leioa, Spain; b Central Analysis Service, Faculty of Science and Technology, University of the Basque Country UPV/EHU, Leioa, Spain

**Keywords:** Hepatocarcinoma, Phospholipid composition, Mass spectrometry

## Abstract

The data contain information related to the research article entitled “Profiling of promoter occupancy by the SND1 transcriptional coactivator identifies downstream glycerolipid metabolic genes involved in TNFα response in human hepatoma cells” (DOI: 10.1093/nar/gkv858). In the article alluded to, we reported that tumor necrosis factor alpha (TNFα) increases notably the cellular content of the major glycerolipid phosphatidylcholine (PC). Here, accompanying lipidomic data determine the PC structural variants that have been identified in human hepatoma HepG2 cells and those whose relative abundance is modified by TNFα. We used ultrahigh performance liquid chromatography (UHPLC) coupled to electrospray ionization (ESI) tandem mass spectrometry (MS/MS)-based lipidomic profiling to analyze lipid extracts of control and TNFα-treated HepG2 cells. The identity of PC individual species was elucidated using the values of the retention time and molecular weight in addition to the fragmentation patterns. MS data were then processed and analyzed for the characterization of statistically significant differences in detected structural variants. We have annotated the dataset of PC species that characterize HepG2 cells' phenotype, both under normal and pro-inflammatory conditions.

**Table:** Specifications Table

Subject area	*Medicine and Dentistry.*
More specific subject area	*Hepatology.*
Type of data	*Tables (2) and Figures (3).*
How data was acquired	*Mass spectrometry of cell lipid extracts by UHPLC-MS*^*E*^*analysis.* - *Ultrahigh performance liquid chromatographer (UHPLC), Acquity UHPLC*^*TM*^ *system from Waters (Milford, MA, USA) and reverse phase column (Acquity UHPLC HSS T3 1.*8 μm*, 100* × *2.*1 mm*) and precolumn (Acquity UHPLC HSS T3 1.*8 μm *VanGuard).* - *Tandem mass spectrometer quadrupole time of flight (Q-TOF), SYNAPT G2 HDMS (Waters (Milford, MA, USA).*
Data format	*Raw and analyzed* - *Software:* *MassLynx software Version 4.1 (Waters MS Technologies, Manchester, UK).* *MS*^*E*^ *Data Viewer (Waters MS Technologies, Manchester, UK).* *SimLipid 5 software (Premier Biosoft, USA).* *SPSS Statistics Version 24 (IBM, Armonk, NY, USA).*
Experimental factors	*Cancer cell culture, lipid extraction and PC variants' analysis by UHPLC-MS*^*E*^*-based lipidomics.*
Experimental features	*Human hepatoma HepG2 cells were cultured in the presence or the absence of TNFα, then lysed and protein determined. Lipids were extracted from cell lysates using organic solvents following standard procedures and the lipid extracts were submitted to the analysis core facility of the University for PC molecular species identification and quantification.*
Data source location	*Lipids & Liver Research Group, Department of Physiology, Faculty of Medicine and Nursing, University of the Basque Country UPV/EHU, Leioa, Spain.*
Data accessibility	*The data are available in this article.*
Related research article	“Profiling of promoter occupancy by the SND1 transcriptional coactivator identifies downstream glycerolipid metabolic genes involved in TNFα response in human hepatoma cells”, (DOI: 10.1093/nar/gkv858).

**Table:** 

**Value of the Data** • These data facilitate a guidance for further insights into the environment dependency of the human cancer cells lipid code. • This analysis is an example of how lipidomics helps to uncover perturbations of individual lipid species that, while blinded by a global quantitative approach, may be relevant to intracellular signaling and cell function. • The phosphatidylcholine structural variability found in hepatocarcinoma cells might serve as potential biomarker for cancer-associated inflammation.

## 1 Data

**Fig. 1:**
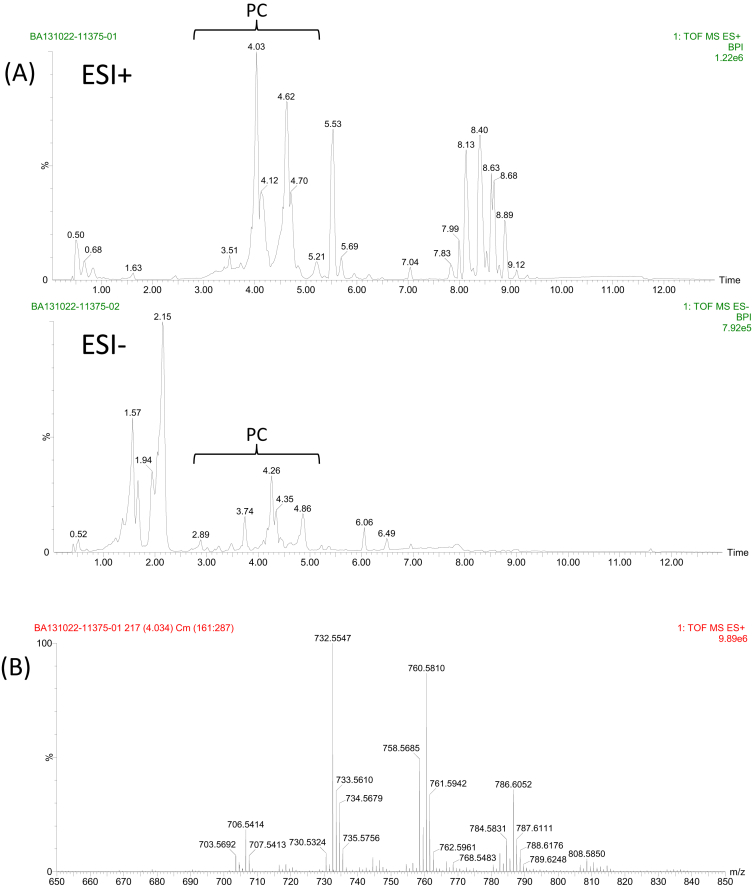
UHPLC-ESI-MS^E^ base peak ion intensity chromatogram in positive- and negative-ion mode (A) and positive mass spectrum for mass range *m/z* 650–850 (B). Approximate retention time regions corresponding to the identified phosphatidylcholine (PC) structural variants are indicated on the plot.

**Fig. 2:**
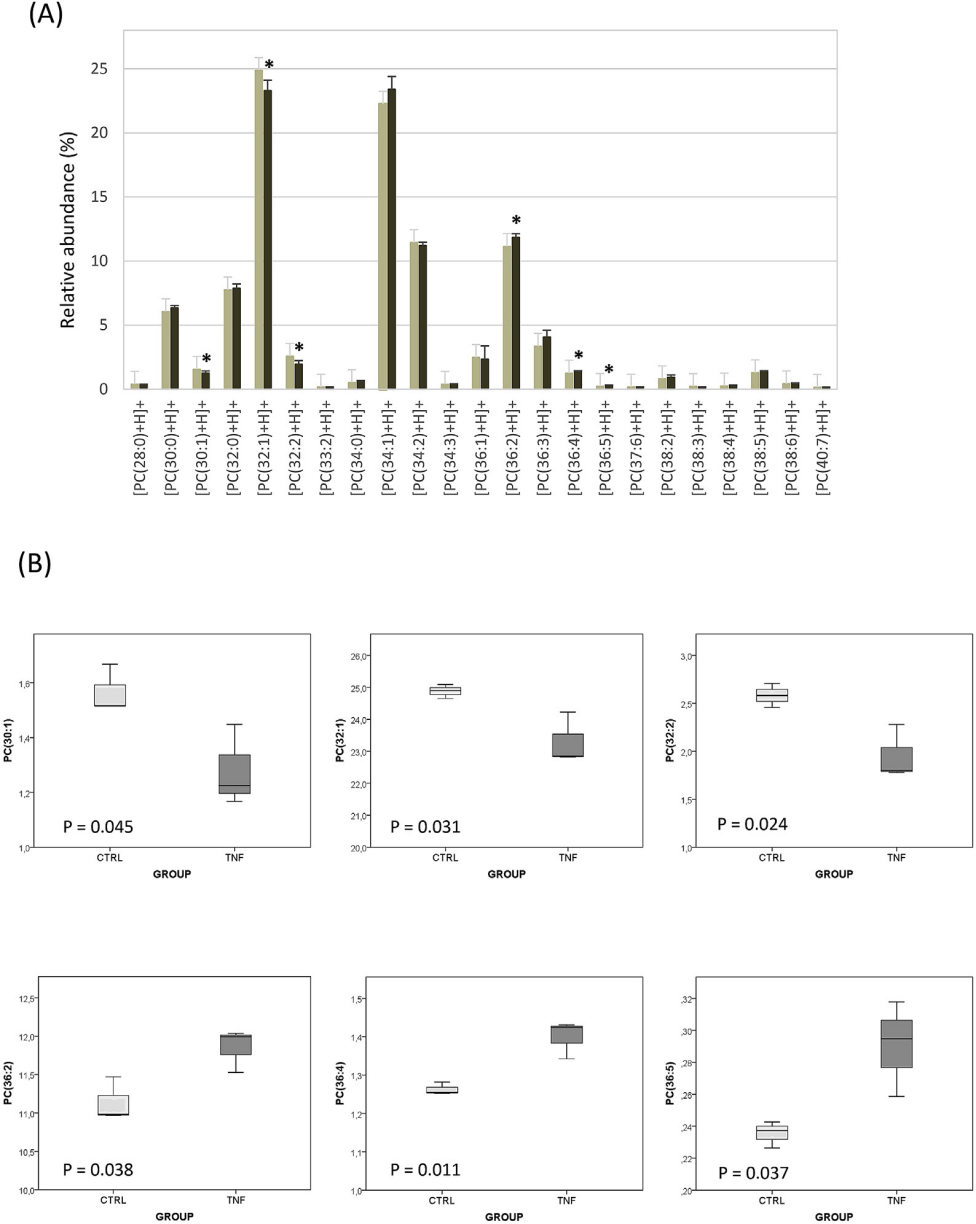
Relative abundance (%) of individual phosphatidylcholine PC structural variants in control and TNFα-treated HepG2 cells: A) determined using relative abundances of [M + H]+ ions in the positive ion HPLC-ESI-MS/MS. Data are expressed as means ± SD. Statistical differences between control (pale gray) and TNFα-treated (dark gray) groups according to *t*-test are denoted by * *P* ≤ 0.05. B) Box-Whisker plots describing the down-regulated and up-regulated species by the treatment.

The Tables and Figures provided in this Data in Brief article gather the raw data of individual phosphatidylcholine (PC) species that have been identified in total lipid extracts of HepG2 cells and highlight those whose relative abundance is modulated by TNFα. Acute TNFα treatment of HepG2 cells is known to increase total cellular PC [1]. The methodology here adopted, tandem mass spectrometry (MS/MS) preceded by a UHPLC separation step, uncovers the heterogeneity in PC species enabling the identification of PC molecular variants at the level of the fatty acids that are bound through an ester bond to the *sn-1* and *sn-2* position of the glycerol backbone. The most intense ions detected for PC are in ESI (electrospray ionization) in the positive-ion mode (ESI+, Fig. 1A); however, relevant MS data from both ESI+ and ESI- analysis have been used for a proper assignment. The set of individual PC species detected in HepG2 cells together with their peak markers (retention time and *m/z* value) and structure are annotated in Table 1. The table includes the raw data values of all experiments performed. In addition to the highly intense protonated forms, which range from [PC(28:0)+H]+ to [PC(40:7)+H]+, some PC molecular variants can be also found as sodium adducts. Given that their abundance is very low and that it is agreed they introduce overfitting and statistical noise [2], the sodium adducts were not taken into account for the compositional estimation. Hence, individual PC species are described using relative abundances (%) of the protonated molecules [M + H]+ in the positive-ion mass spectra obtained by the peak integration of all PCs (Fig. 1B). We annotate in Table 2 the identity and relative abundance of the protonated variants detected both in control and in TNFα-treated HepG2 cells. Fig. 2A shows clearly that the composition of PC in terms of the number of carbons and unsaturations of fatty acyls is highly heterogeneous. Interestingly, two species, PC(32:1) or PC(16:0/16:1) and PC(34:1) or PC(16:0/18:1), account for nearly 50% of the total intensity while more than ten species exhibit a representation lower than 1%. Statistical analysis of the differences between groups revealed that PC synthesis was not globally up-regulated by TNFα as there was a significant change in the relative abundance of six out of the 23 individual species examined according to the *t*-test (numerical *P* values are listed in Table 2 and included in Fig. 2B). Three species were up-regulated, namely PC(36:2), PC(36:4) and PC(36:5), and an opposite trend was seen in PC(30:1), PC(32:1) and PC(32:2), (data are shown as Box-Whisker plots in Fig. 2B). The absolute quantitative data (in nmol/mg protein) of individual lipid species may be calculated as their relative abundances multiplied by the total PC concentration in the corresponding sample. In parallel experiments aimed at replicating those reported previously [1], we measured PC mass in aliquots of the lipid extracts [3]. We found that it averaged 27.20 ± 1.23 nmol/mg protein in the control and 32.69 ± 3.27 nmol/mg protein in the treated group (mean ± SD), indicating that TNFα increased the absolute PC mass in HepG2 cells by an extent (20.18%) similar to that earlier found [1]. For those readers that are not familiar with this type of lipidomic approach, it is important to outline that present data provide the identification of PC structural variants whose TNFα-promoted increase is above or below the average 20.18% rise. In other words, present dataset enables the identification of prevalent combinations of fatty acids in the PC class of phospholipids. Remarkably, pro-inflammatory TNFα seems to favor the contribution of PC species containing di- and polyunsaturated fatty acids (like eicosanoids) in detriment of less unsaturated fatty acid-containing species.

**Table 1:** Phosphatidylcholine (PC) individual species and their corresponding peak markers (mass to charge ratio (*m/z*) and retention time (RT) pairs) detected in HepG2 cells. E, experiment. The PC specific structure at the level of the fatty acid bound to the *sn-1* and *sn-2* position of the glycerol backbone was assigned as described in the Experimental Design, Materials, and Methods section.

Species	*m/z* [PC + H]^+^ or [PC + Na]^+^			RT (min)			#Carbon	#Double Bond	Structure
	E1	E2	E3	E1	E2	E3			
[PC(28:0)+H]+	678.5066	678.5070	678.5070	3.30	3.30	3.30	28	0	[PC(14:0/14:0)+H]+
[PC(30:0)+Na]+	728.5193	728.5226	728.5197	3.94	3.95	3.94	30	0	[PC(16:0/14:0)+Na]+
[PC(30:1)+H]+	704.5228	704.5230	704.5223	3.41	3.41	3.40	30	1	[PC(14:0/16:1)+H]+
[PC(32:0)+H]+	734.5710	734.5707	734.5702	4.55	4.55	4.55	32	0	[PC(16:0/16:0)+H]+
[PC(32:0)+Na]+	756.5524	756.5518	756.5520	4.55	4.55	4.55	32	0	[PC(16:0/16:0)+Na]+
[PC(32:1)+H]+	732.5537	732.5563	732.5541	4.04	4.04	4.04	32	1	[PC(16:0/16:1)+H]+
[PC(32:1)+Na]+	754.5346	754.5378	754.5365	4.03	4.04	4.04	32	1	[PC(16:0/16:1)+Na]+
[PC(32:2)+H]+	730.5353	730.5394	730.5394	3.51	3.51	3.51	32	2	[PC(14:0/18:2)+H]+
[PC(32:2)+Na]+	752.5163	752.5201	752.5215	3.51	3.51	3.51	32	2	[PC(14:0/18:2)+Na]+
[PC(33:2)+H]+	744.5534	744.5551	744.5534	3.82	3.82	3.82	33	2	[PC(16:0/17:2)+H]+
[PC(34:0)+H]+	762.6032	762.6020	762.6018	5.17	5.17	5.17	34	0	[PC(16:0/18:0)+H]+
[PC(34:1)+H]+	760.5860	760.5853	760.5864	4.63	4.63	4.63	34	1	[PC(16:0/18:1)+H]+
[PC(34:1)+Na]+	782.5678	782.5669	782.5675	4.63	4.63	4.63	34	1	[PC(16:0/18:1)+Na]+
[PC(34:2)+H]+	758.5678	758.5706	758.5704	4.14	4.14	4.14	34	2	[PC(16:0/18:2)+H]+
[PC(34:2)+Na]+	780.5490	780.5534	780.5522	4.19	4.21	4.14	34	2	[PC(16:0/18:2)+Na]+
[PC(34:3)+H]+	756.5534	756.5553	756.5549	3.68	3.68	3.68	34	3	[PC(16:0/18:3)+H]+
[PC(36:1)+H]+	788.6183	788.6160	788.6167	5.22	5.22	5.22	36	1	[PC(18:0/18:1)+H]+ or [PC(16:0/20:1)
[PC(36:1)+Na]+	810.6002	810.5983	810.5984	5.21	5.22	5.22	36	1	[PC(18:0/18:1)+Na]+ or [PC(16:0/20:1)+Na]+
[PC(36:2)+H]+	786.6017	786.6011	786.6015	4.71	4.71	4.71	36	2	[PC(18:0/18:2)+H]+
[PC(36:2)+Na]+	808.5825	808.5826	808.5837	4.71	4.72	4.72	36	2	[PC(18:0/18:2)+Na]+
[PC(36:3)+H]+	784.5838	784.5867	784.5860	4.26	4.26	4.26	36	3	[PC(16:0/20:3)+H]+
[PC(36:3)+Na]+	806.5664	806.5695	806.5683	4.25	4.25	4.24	36	3	[PC(16:0/20:3)+Na]+
[PC(36:4)+H]+	782.5663	782.5714	782.5698	4.11	4.11	4.11	36	4	[PC(16:0/20:4)+H]+
[PC(36:4)+H]+	782.5669	782.5692	782.5690	3.74	3.74	3.73	36	4	[PC(16:0120:3)+H]+
[PC(36:5)+H]+	780.5533	780.5551	780.5538	3.70	3.70	3.70	36	5	[PC(16:1/20:4)+H]+
[PC(37:6)+H]+	792.5545	792.5544	792.5548	4.71	4.71	4.71	37	6	[PC(17:2/20:4)+H]+
[PC(38:2)+H]+	814.6348	814.6336	814.6340	5.26	5.26	5.26	38	2	[PC(18:0/20:2)+H]+
[PC(38:2)+Na]+	836.6156	836.6150	836.6145	5.26	5.27	5.28	38	2	[PC(18:0/20:2)+Na]+
[PC(38:3)+H]+	812.6191	812.6189	812.6174	4.97	4.97	4.84	38	3	[PC(18:0/20:3)+H]+
[PC(38:4)+H]+	810.6000	810.5995	810.5994	4.73	4.73	4.73	38	4	[PC(18:0/20:4)+H]+
[PC(38:4)+H]+	810.6002	810.5983	810.5984	5.21	5.22	5.22	38	4	[PC(16:0/22:4)+H]+
[PC(38:5)+H]+	808.5830	808.5868	808.5861	4.18	4.18	4.18	38	5	[PC(16:0/22:5)+H]+
[PC(38:6)+H]+	806.5677	806.5705	806.5705	3.75	3.76	3.75	38	6	[PC(16:0/22:6)+H]+
[PC(38:6)+H]+	806.5683	806.5718	806.5690	3.94	3.94	3.94	38	6	[PC(16:0/22:6)+H]+
[PC(40:7)+H]+	832.5844	832.5870	832.5830	4.00	4.00	4.00	40	7	[PC(18:1/22:6)+H]+

**Table 2:** Relative abundance (%) of individual phosphatidylcholine (PC) species in control and TNFα-treated human hepatoma HepG2 cells. An unpaired Student's t-test was carried out to compare values in control and treated cells (SPSS Statistics version 24, IBM, Armonk, NY, USA). SD, standard deviation. E, experiment. Statistical significance is defined as *P* ≤ 0.05.

Species	RT (min)	*m*/*z*	Control HepG2 cells					TNFα-treated HepG2 cells					P value	# Carbon	#Double Bond
			% of total intensity					% of total intensity							
			E1	E2	E3	Mean	SD	E1	E2	E3	Mean	SD			
[PC(28:0)+H]+	3.30	678.507	0.414	0.409	0.378	0.400	0.020	0.408	0.345	0.354	0.369	0.034	0.240	28	0
[PC(30:0)+H]+	3.94	706.538	5.990	6.059	6.086	6.045	0.049	6.124	6.424	6.483	6.343	0.192	0.060	30	0
[PC(30:1)+H]+	3.40	704.523	1.516	1.515	1.668	1.566	0.088	1.448	1.167	1.226	1.280	0.148	0.045	30	1
[PC(32:0)+H]+	4.55	734.571	8.242	7.605	7.389	7.745	0.443	7.568	8.243	7.801	7.871	0.343	0.718	32	0
[PC(32:1)+H]+	4.04	732.554	24.898	25.086	24.648	24.877	0.219	24.228	22.816	22.849	23.298	0.806	0.031	32	1
[PC(32:2)+H]+	3.51	730.535	2.707	2.582	2.458	2.582	0.124	2.280	1.780	1.801	1.954	0.283	0.024	32	2
[PC(33:2)+H]+	3.82	744.553	0.175	0.169	0.170	0.171	0.003	0.184	0.166	0.149	0.166	0.017	0.631	33	2
[PC(34:0)+H]+	5.17	762.603	0.589	0.544	0.437	0.523	0.078	0.694	0.619	0.574	0.629	0.060	0.136	34	0
[PC(34:1)+H]+	4.63	760.586	22.061	22.130	22.525	22.239	0.250	22.305	24.293	23.574	23.391	1.006	0.127	34	1
[PC(34:2)+H]+	4.14	758.568	11.047	11.647	11.618	11.437	0.338	11.421	10.944	11.282	11.215	0.245	0.410	34	2
[PC(34:3)+H]+	3.68	756.553	0.363	0.415	0.401	0.393	0.027	0.430	0.378	0.359	0.389	0.037	0.899	34	3
[PC(36:1)+H]+	5.22	788.618	2.412	2.567	2.460	2.479	0.079	1.140	3.017	2.877	2.344	1.046	0.834	36	1
[PC(36:2)+H]+	4.71	786.602	10.968	10.980	11.472	11.140	0.288	11.528	12.037	11.993	11.853	0.282	0.038	36	2
[PC(36:3)+H]+	4.26	784.584	3.503	3.380	3.188	3.357	0.159	4.372	4.382	3.469	4.074	0.525	0.086	36	3
[PC(36:4)+H]+	4.11	782.566	1.252	1.255	1.282	1.263	0.016	1.431	1.342	1.424	1.399	0.049	0.011	36	4
[PC(36:5)+H]+	3.70	780.553	0.226	0.237	0.243	0.235	0.008	0.318	0.259	0.295	0.290	0.030	0.037	36	5
[PC(37:6)+H]+	4.71	792.555	0.174	0.171	0.172	0.172	0.002	0.169	0.165	0.180	0.171	0.008	0.837	37	6
[PC(38:2)+H]+	5.26	814.635	0.875	0.824	0.772	0.824	0.051	1.140	0.860	0.798	0.932	0.182	0.376	38	2
[PC(38:3)+H]+	4.97	812.619	0.117	0.118	0.434	0.223	0.183	0.174	0.039	0.129	0.114	0.068	0.389	38	3
[PC(38:4)+H]+	5.22	810.600	0.274	0.267	0.248	0.263	0.013	0.322	0.292	0.282	0.298	0.021	0.067	38	4
[PC(38:5)+H]+	4.18	808.583	1.328	1.359	1.192	1.293	0.089	1.428	1.391	1.258	1.359	0.089	0.417	38	5
[PC(38:6)+H]+	3.94	806.568	0.466	0.450	0.401	0.439	0.034	0.500	0.437	0.455	0.464	0.033	0.411	38	6
[PC(40:7)+H]+	4.00	832.584	0.160	0.148	0.152	0.153	0.006	0.151	0.172	0.170	0.165	0.011	0.209	40	7

## 2 Experimental design, materials, and methods

### 2.1 Cell culture and lipid extraction

HepG2 cells (ATCC) (3.5 × 10^6^) were seeded in 10 cm diameter plates and grown in Eagle's minimal essential medium (ATCC) supplemented with 2 mM L-glutamine, 100 IU/ml penicillin, 100 μg/ml streptomycin (all from Sigma-Aldrich) and 10% (v/v) fetal bovine serum (ATCC) at 37 °C and 5% CO_2_. Cultures were either left untreated or treated with TNFα (50 ng/ml) for 24 h before harvesting as described [1]. Cells were then lysed and lipids were exhaustively extracted from cell lysates with CHCl_3_/MeOH [4], dried in a Savant SpeedVac concentrator, and stored at −80 °C under N_2_ atmosphere prior to their injection into the UHPLC-MS/MS system. Aliquots of lipid extracts were analyzed for the content of total PC using our developed thin–layer-chromatography/optical densitometry method [3].

### 2.2 UHPLC-MS/MS analysis

Ultrahigh performance liquid chromatography was carried out using an ACQUITY UHPLC system from Waters (Milford, MA, USA) equipped with a binary solvent delivery pump, an autosampler and a column oven. Separation of the PC structural variants was performed with Acquity UHPLC HSS T3 column (100 × 2.1 mm, 1.8 μm) and precolumn (VanGuard, 1.8 μm) as thoroughly described elsewhere [5]. All UHPLC-MS^E^ data were acquired on a SYNAPT G2 HDMS, with a quadrupole time of flight (Q-TOF) configuration (Waters, Milford, MA, USA) equipped with an ESI source operated in positive or negative ion mode. The mass spectrometer was operated in the continuum MS^E^ acquisition mode for both polarities. During this acquisition method, the first quadrupole is operated in a wide band rf mode only, allowing all ions to enter the T-wave collision cell. Two discrete and independent interleaved acquisition functions were automatically created: the first function, typically set at 6 eV, collected low energy or unfragmented data, whereas the second function collected high energy or fragmented data typically obtained by using a collision energy ramp from 15–40 eV. The other MS parameters were described previously in Ref. [6]. Representative chromatograms in the positive- and in the negative-ion mode are shown in Fig. 1A and an example of PC mass spectra in Fig. 1B.

### 2.3 Lipid identification and MS data processing

**Fig. 3:**
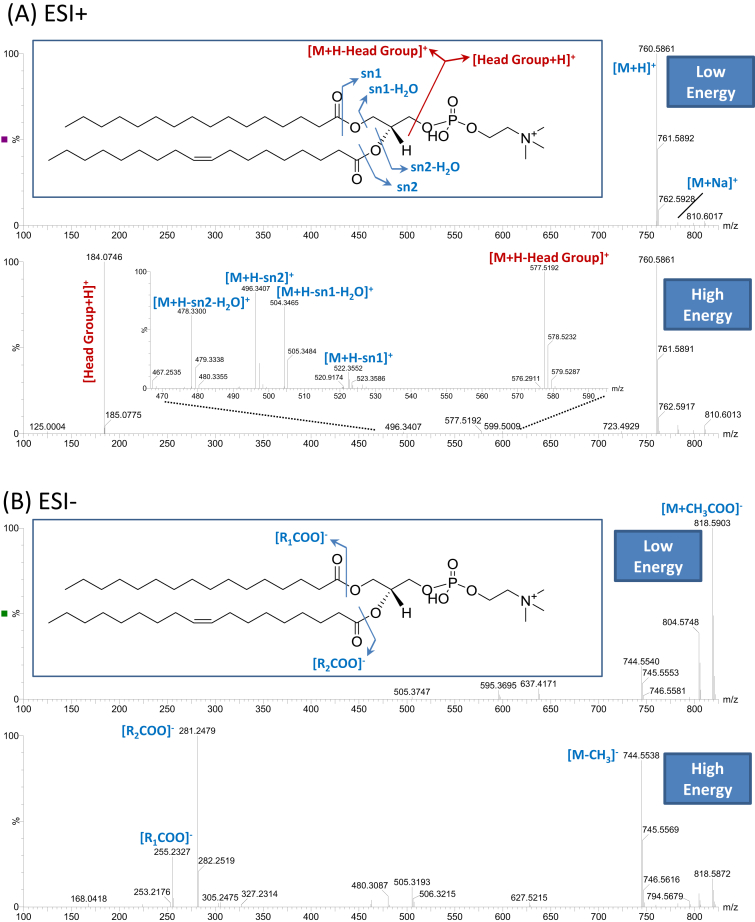
Chemical structure and fragmentation pattern of PC(16:0/18:1). Low and high collision energy mass spectra of PC(16:0/18:1) during MS^E^ experiment in polarity mode ESI+ (A) and ESI- (B).

For lipid identification, the data generated by the UHPLC-MS^E^ analysis in positive and negative ion mode were extracted using MS^E^ data viewer (Waters MS Technologies, Manchester, UK) generating an exportable text file, which was used for lipid identification using SimLipid software (Premier Biosoft, USA). This software matches the exact masses of the precursor and product ions of unknown lipids with those on an *in-silico* database containing over 40,298 lipid species and 1,509,305 structure specific MS/MS characteristic ions. SimLipid assigns a probability score to the unknown lipid structure according to the best fit of experimental *m*/*z* values with theoretical *m*/*z* values of both precursor and product ions of the SimLipid database. By matching the exact masses of the characteristic fragment ions, in addition to precursor ions, SimLipid was able to identify isomers with similar *m*/*z*. Tolerance for MS and MS/MS used for identification was 5 mDa. Then, diagnostic fragments of the polar head group or the fatty acyls chains were investigated to confirm the annotation proposed by the database and discriminate isomers. Fig. 3 illustrates the chemical structure and fragmentation pattern of PC(16:0/18:1) during our MS^E^ analyses. In addition to this, elution order was used to minimize misidentification. Within the PC class, retention time decreases with the number of double bonds and increases with the length of the acyl chains. Lipids that did not meet these two conditions were considered false positives.

For MS data processing and, in order to convert the three-dimensional LC–MS raw data (RT, *m*/*z*, intensity) into a table of time-aligned detected features, with their RT, *m*/*z*, and intensity in each sample, data were acquired with the MassLynx V4.1 software and processed using the Databridge 3.5 converter (Waters, Milford, USA), XCMS 1.42.0 (Metlin, La Jolla, CA, USA), as reported previously [6]. Individual PC species were quantitated using relative abundances (%) of the protonated molecules [M + H]+ in the positive-ion mass spectra obtained by the peak integration of all PCs. The final dataset were exported into SPSS V20 for statistical analysis.

### 2.4 Statistical analyses

Data are reported as the mean ± SD and an unpaired Student's t-test was carried out to compare control and TNFα-treated groups (SPSS Statistics version 24, IBM, Armonk, NY, USA). Statistical significance is defined as *P* ≤ 0.05.

## Funding

This work was supported by the Basque Government Departments of Education (grant IT971-16) and Economic Promotion (grant KK2018/00090), Basque Country, Spain.
